# The Impact of Baseline Mindfulness Scores on Mindfulness-Based Intervention Outcomes: Toward Personalized Mental Health Interventions

**DOI:** 10.3389/fpsyg.2022.934614

**Published:** 2022-07-11

**Authors:** Rodrigo C. Vergara, Constanza Baquedano, Enrique Lorca-Ponce, Christoph Steinebach, Álvaro I. Langer

**Affiliations:** ^1^Departamento de Kinesiología, Facultad de Artes y Educación Física, Universidad Metropolitana de Ciencias de la Educación, Santiago, Chile; ^2^Centro Nacional de Inteligencia Artificial, Santiago, Chile; ^3^Center for Social and Cognitive Neuroscience, School of Psychology, Universidad Adolfo Ibañez, Santiago, Chile; ^4^Faculty of Medicine, School of Nursing, Universidad Finis Terrae, Santiago, Chile; ^5^School of Applied Psychology, ZHAW Zürich University of Applied Sciences, Zurich, Switzerland; ^6^Mind-Body Lab, Faculty of Medicine, Institute of Psychological Studies, Universidad Austral de Chile, Valdivia, Chile; ^7^Millennium Nucleus to Improve the Mental Health of Adolescents and Youths, Santiago, Chile

**Keywords:** baseline, mindfulness-based interventions, personal trajectories, RCTs outcomes, mental health

## Abstract

A growing body of evidence has portrayed mindfulness as a useful tool for dealing with a broad range of psychological problems and disorders. This has created the impression that mindfulness-based interventions (MBIs) can be used to treat nearly all psychological difficulties, in all cases. Nonetheless, little research has been done on how individual differences may contribute to intervention outcomes. The goal of this study was to evaluate the role of baseline mindfulness on participants’ outcomes by examining three prior Randomized Controlled Trials that addressed the impact of MBIs on mental health and mindfulness measures. The participants were 164 people, aged between 12 and 45, from both clinical and non-clinical samples. Our findings indicate that at least two thirds of the change produced by these interventions in terms of mindfulness scores can be predicted by the baseline scores of the same variables. We also found that many trajectories are not only strongly influenced by the initial status of the participants, but also by the intervention performed, as attested to by the significant interactions found. These results stress the need to continue doing research in a way that considers the diversity of participants’ trajectories, increasing the room for intervention improvements aligned with a more personalized health care model.

## Introduction

Meditation has been defined as “a family of complex emotional and attentional regulatory strategies developed for various purposes, among them the cultivation of well-being and emotional balance” (Lutz et al., 2008). Even though meditation is originated in the Eastern culture, in recent decades it has been widely practiced in the West, where it has been adopted as a way of improving the quality of life. Many secular practices have been derived from it, mainly oriented toward addressing specific mental health problems in modern society, such as stress, anxiety and depression (Dakwar and Levin, 2009).

In the West, mindfulness meditation is the most popular and scientifically studied meditation technique. Mindfulness has been defined from a scientific perspective, on an operational level, as “paying attention in a particular way: on purpose, in the present moment and non-judgmentally” (Kabat-Zinn, 1994). By maintaining this non-elaborative stance, the ongoing flow of sensory, cognitive and affective events which arise in the mind are acknowledged and accepted as they are (Bishop, 2004; Lutz et al., 2005). A mindful state of mind is an inherent capacity of human being, that could be present as a trait, that is to say, as an everyday life disposition or stable tendency to be mindful (Brown and Ryan, 2003). This Mindfulness disposition can be further developed or enhanced through the practice of several meditation techniques (Ricard et al., 2014).

Among others forms, mindfulness can be cultivated through structured courses (usually composed of weekly group sessions) where contemplative skills (e.g., attentional training) are taught and discussed. The interventions which are based on the formal training of mindfulness (e.g., Mindfulness-Based Stress Reduction [MBSR] or Mindfulness Based Cognitive Therapy [MBCT]) have been label as mindfulness-based interventions (MBIs) (Howarth et al., 2019). Over the last 10 years, mounting evidence has presented mindfulness as a useful tool for dealing with a number of psychological problems (e.g., stress, anxiety, depression) (Shonin et al., 2013) and also as an intervention capable of fostering attentional and emotion regulation qualities (Tang et al., 2015). This has created the impression that mindfulness is useful for treating almost all psychological difficulties, for everyone. Nonetheless, little research has been done on how individual differences may contribute to intervention outcomes. Considering that averages may hide potentially hazardous trajectories, and that mindfulness researchers may have failed to report adverse effects of interventions (Joiner, 2017; Britton, 2019), it is critical to assess individual trajectories considering certain attributes measured before intervention.

In the last 5 years, a considerable number of studies on MBIs have reported negative findings when assessing their effects (Britton, 2019). These negative findings may be derived from differences in construct operationalization, issues with control group set-up, or inadequate cultural measurements (Davidson and Kaszniak, 2015). Even the role of individual differences such as personality traits (Harnett et al., 2016) and mindfulness measured prior to intervention (Tortella-Feliu et al., 2020) may play a relevant role in the results of Randomized Controlled Trials (RCTs) of MBIs. For instance, participants with an insecure attachment style benefit more from mindfulness-based stress reduction (MBSR) programs than participants with a secure attachment (Cordon et al., 2009). People’s empathy scores predict preferences for loving-kindness, with females tending to prefer loving-kindness more than males (Tang and Braver, 2020). Likewise, non-reactivity and non-judgment of present moment experiences have been found to predict a preference for engaging in open monitoring (Tang and Braver, 2020). Personality differences even explain preferences for specific MBSR techniques (Barkan et al., 2016), which may impact autonomous work and therefore intervention trajectories.

One central aspect which has been neglected is the impact of pre-intervention mindfulness scores, even though some correlational studies have shown a relation between mindfulness scores and psychological distress regulation, depression, anxiety, and stress (Shapiro et al., 2011; Harnett et al., 2016; Tortella-Feliu et al., 2020). This suggests that mindfulness scores prior to an intervention may place participants into different trajectories as a result of the regulation tools that they may use. Nonetheless, experimental studies have yielded contradictory evidence, with some reporting a relevant impact of baseline mindfulness on intervention outcome (Shapiro et al., 2011) and others finding negative or weak results (Tortella-Feliu et al., 2020).

The contradictory results found in the literature can be due to a number of aspects. For example, it is worth pointing out that these assessments did not evaluate the interaction between baseline mindfulness and the tested intervention, which may mask positive results. Also, these studies used baseline mindfulness to predict depression, anxiety, and stress, when in causal terms it is the change in mindfulness due to the intervention that is expected to produce an impact on these variables. Above all, it is also necessary to consider the differences between the instruments used to measure mindfulness as well as sample disparities, all of which may contribute to these discrepancies.

Given that research assessing the impact of baseline mindfulness on participants’ trajectories during mindfulness interventions is scarce and non-conclusive, the goal of this study is to evaluate the role of baseline mindfulness on participants’ outcomes taking into account previous RCTs of mindfulness and the potential limitations described above (i.e., interaction evaluation, considering mindfulness change as a source of outcome, different mindfulness instruments, and different populations). The results derived from this reassessment of RCTs will contribute to the development of mindfulness interventions adapted to particular group or even individual needs rather than the blind application of the intervention neglecting group characteristics and needs.

## Materials and Methods

### Participants and Design

In this study, we utilized three samples from previous studies that involved MBIs, as well as RCT designs from our research group. 1:1 allocation was used in a simple randomization process (Langer et al., 2010, 2017, 2020). The three samples featured 164 participants aged 12–45 from clinical and non-clinical populations. The control groups were as follows: for University Students (University-MBCT), we used a cinema-forum as an active control group; for School Students (School-MiSP), Education as usual (EAU) was used; and for the first psychotic episode patients, treatment as usual (TAU), standard psychopharmacology, and psychosocial treatments were used (Psychosis-MBCT). The samples were heterogeneous in terms of the percentage of men and women in each study. The age averages correspond to an adolescents’ sample and two samples of young adults. The samples were also diverse in relation to the context in which they were applied. In turn, the studies were implemented at different levels of prevention (i.e., universal and targeted) and treatment (i.e., early intervention) (see Table 1 for details).

**TABLE 1:** Description of RCTs.

Sample name	Study focus and country	Sample profile (*n*)	Age (*SD*) male %	Instruments	Intervention and control
University-MBCT	Targeted prevention Spain	Undergraduate students with distressing HLE (*n* = 38)	21.31 (2.58) 15.8%	BDI-I AAQ-II	Adapted MBCT vs. Active control group
School-MiSP	Universal prevention Chile	School students (*n* = 88)	13.37 (0.57) 47.72%	DASS-21 CAMM	MiSP vs. EAU (waiting list)
Psychosis-MBCT	Early Intervention Chile	Patients with psychosis (*n* = 38)	23.8 (4.82) 78.9%	DASS-21 FFMQ	Adapted MBCT vs. TAU (waiting list)

*Characteristics of participants and studies.*

Among the reasons for testing MBIs from an RCT was to test their effect in populations (Psychosis), social contexts (Chile) or experiences (Hallucinations Like experiences, HLEs) with scarce research. Thus, the overall objective for selecting MBIs in relation to other interventions was to provide participants with mind/body strategies that enable them to establish a different relationship with their internal stressful events, thus achieving greater psychological flexibility and a more harmonious relationship with themselves and their peers. Another relevant background for the selection of the MBIs was that they have proven to be well received by adolescents and young people (e.g., Monshat et al., 2013).

### Instruments

For Mental Health, the Beck Depression Inventory (BDI) (Beck et al., 1988) has 21 items on depression symptoms experienced over the last 2 weeks. Answers to each item are presented as a 4-point Likert scale from 0 (I do not feel sad) to 3 (I am so sad and unhappy that I cannot stand it). Excellent internal consistency has been reported in Spanish-speaking adolescents (Cronbach’s α = 0.92) (del Beltrán et al., 2012).

Depression, Anxiety and Stress scale (DASS-21; Lovibond and Lovibond, 1995). In this study, we used the Chilean validation (Antúnez and Vinet, 2012), whose reliability is adequate (Cronbach’s α = 0.91). This scale is made up of twenty-one items assessing symptoms of depression (seven items), anxiety (seven items), and stress (seven items). Responses are recorded on a scale ranging from 0 (“It didn’t happen to me”) to 3 (“It happened to me a lot, or most of the time”).

For Mindfulness, the Acceptance and Action Questionnaire-II (AAQ-II; Bond et al., 2011), a widely used instrument for assessing Experiential Avoidance (EA; Hayes et al., 1996). EA can be defined as a person’s attempts or desires to suppress unwanted internal experiences like thoughts, emotions, memories, or bodily sensations (Hayes et al., 1996). This is a seven-item self-administered scale with seven-point Likert-type response options from 1 (never) to 7 (always). A higher AAQ-II total score indicates a higher level of experiential avoidance. The AAQ-II has been shown to have a unifactorial internal structure (Bond et al., 2011) and has been satisfactorily adapted to multiple cultural contexts and populations [e.g., Greece (Karekla and Michaelides, 2017), Malaysia (Shari et al., 2019), Turkey (Yavuz et al., 2016), China (Zhang et al., 2014), Serbia (Zuljevic et al., 2020)]. In this study, we used the Spanish version of the AAQ-II (Ruiz et al., 2013).

Five Facet Mindfulness Questionnaire (FFMQ; Baer et al., 2006). This is a self-reporting questionnaire that describes mindfulness operationally as a multidimensional construct, built on the following five facets: observing, describing, acting with awareness, non-judging of experiences, and non-reactivity to experience. The Spanish version used in this study (Schmidt and Vinet, 2015) exhibits acceptable to good levels of reliability (Cronbach’s α = 0.62–0.86).

Child and Adolescent Mindfulness Measure (CAMM). The CAMM (Greco et al., 2011) comprises 10 items and a five-point Likert scale ranging from 0 (never true) to 4 (always true), which are used to evaluate mindfulness skills. Greco’s original scale has adequate internal consistency (Cronbach’s α = 0.81) and construct validity. We used the seven-item Spanish version of the CAMM, which has been shown to be more valid and reliable than the 10-item version (García-Rubio et al., 2019).

### Intervention

In the first (University students) and third sample (patients with psychosis), we used a reduced or less intense version of the MBCT. In particular, we maintained the structure of each session, but the length of the session and the mindful practices were reduced. The length of each session was approximately 1 h and a half. The meditation practice lengths did not exceed 20–25 min in university students and 10–12 min in patients with psychosis. The interventions included exercises such as guided body scan, sitting and walking meditation, gentle stretching, intentional attention to body sensations, thoughts, and feelings, and take-home exercises. Additionally, every participant received a flash drive or CD with guided mindfulness practice audio recordings and a booklet with the contents of each session.

The “.b curriculum” from the Mindfulness in Schools Project (MiSP; Mindful Nation UK, 2015) was implemented in the second sample (school students) using a workshop format of eight weekly sessions lasting 45 min each. All sessions were conducted during normal school hours and in the students’ usual classrooms. During the program (following the MBSR approach), each workshop session was developed around a central theme, making use of specific visual learning aids (slides). In each session, both formal and informal mindfulness exercises are taught. The formal practices are time-limited (approximately 10 min) and are used to train awareness of bodily sensations, emotions, and thoughts. (e.g., body scan, mindful movement, sitting meditation). Informal practices include tooth brushing, mindful eating, and dish-washing, among other activities, which help cultivating present moment awareness in daily life. Moreover, each participant was given a notebook containing a summary of each session and the exercises to be done at home. Audio recordings containing key meditative practices were also provided.

### Ethical Approval

The revised RCT were evaluated by an Ethics committee or an Institutional Review Board and all participants have their consent to participate of the studies. The studies were evaluated as follow: University-MBCT (Doctoral thesis AIL; research group HUM 760, Almeria University), School-MiSP (project n° 82130055: Faculty of Psychology of the Pontificia Universidad Católica de Chile), Psychosis-MBCT (project n° 11150846; National Health Service in Valdivia). The RCT with an available registration number is the Psychosis-MBCT study (ISRCTN24327446).

### Data Analysis

In order to evaluate the impact of baseline mindfulness on individual treatment trajectories, we first determine how baseline mindfulness affects post-intervention mindfulness scores. This analysis allows us to assess the room for mindfulness change given a certain baseline score. Since the room for change may be a consequence of being in the treatment or control group, we also considered an interaction between both variables. Then, to evaluate the impact on psychological health, we used the change between post intervention and baseline mindfulness to predict the same change in depression, anxiety, and stress according to the information provided by each RCT. Given that we are interested in understanding how baseline profiles affect treatment outcome, in this second step we also include the baseline scores of the dependent variable. For instance, if we are predicting a change in depression score, we also use baseline depression as predictor.

We evaluated baseline mindfulness over individual treatment trajectories in two steps using multiple linear regression. For the first step, we used the change in mindfulness measurements (post minus baseline) available in each RCT as dependent variable and baseline mindfulness scores as predictor. We also included intervention (Mindfulness/Control) as independent variable with an interaction with baseline score. For the second step, we use mental health by means of depression, anxiety, and stress change (post minus baseline; according to RCT availability) as dependent variable. As predictors we included the baseline score of the dependent variable, mindfulness change, and the intervention. Given that the FFMQ present many subscales, we only evaluated the interactions of the change in FFMQ subscales with the baseline score once the model was pruned. We did not evaluate interactions between FFMQ subscales. The same analyses reported for post-treatment scores were also performed with the follow-up data using baseline scores as reference. The procedure was performed independently for all three RCTs: University Students (University-MBCT), School Students (School-MiSP), and first psychotic episode patients (Psychosis-MBCT; see Table 1 for details).

All tests were evaluated for multicollinearity using the variance inflation factor (VIF). Considering models without interactions, we regarded variables with a VIF < 3 as independent regressors. If VIF > 3, variables were tested separately and the model with the highest R squared was reported. Models were pruned using the backward method. Finally, for the mental health regression models we reported squared R for the full model reported and only using baseline score of the mental health variable evaluated.

In order to facilitate the contrast with the classic analytic approach we also reported all RCT results using Mixed ANOVA with Measurement (Pre, Post) as within participant’s variable and Group (Control, Mindfulness) as between participants’ variables. We reported averages and standard deviations of all four conditions, sample sizes as well as *p*-values for Group, Measurement, and the interaction. We also included generalized eta squared as effect size index (η^2^G; small: 0.01 – < 0.06; medium: 0.06 - < 0.14; large: ≥ 0.14; range: [0,1]). All analyses were performed using R (R Core Team, 2019). Plots were performed using ggplot2 (Wickham, 2016), linear modeling diagnostics were assessed using car (Fox and Weisberg, 2011), and mixed ANOVAs were performed using ez (Lawrence, 2016).

## Results

### Assessing Classic Randomized Controlled Trial Results

When assessing classic RCT analysis results, we can observe how Psychosis-MBCT and School-MiSP presented significant changes in mindfulness which can be attributed to mindfulness interventions. However, this was not the case for the University-MBCT. When we examine in detail the effects of mindfulness intervention on Psychosis-MBCT (Table 2), we can notice that for all the impacts are detected through interactions. These interactions depict a masked effect over Group variable; while Control group reduces its mindfulness scores post intervention, Mindfulness interventions increase those scores, meaning that the intervention is working as expected on that regard. The same pattern is Observed for the School-MiSP. For the University-MBCT the same pattern is observed, however, the effect is remarkably low, which produced non-significant results.

**TABLE 2:** Mixed ANOVA results for the three RCTs.

Sample	Scale	Subscale	Tau			Mindfulness			Group *p* (η^2^g)	Measurement *p* (η^2^g)	Interaction *p* (η^2^g)
							
			Pre *M* (±SD)	Post *M* (±SD)	n	Pre *M* (±SD)	Post *M* (±SD)	n			
Psychosis MBCT	FFMQ	Observe	27.79 (±5.09)	24.07 (±7.18)	14	25.4 (±5.91)	26.8 (±6.79)	20	0.929 (≈0)	0.521 (0.003)	0.027 (0.041)
		Describe	26.57 (±5.47)	23.64 (±4.18)	14	25.47 (±5.52)	28.05 (±7.02)	19	0.37 (0.021)	0.784 (≈0)	0.004 (0.056)
		Act Aware	23.71 (±7.02)	26.57 (±5.71)	14	23.75 (±4.53)	24.55 (±6.01)	20	0.556 (0.008)	0.148 (0.021)	0.369 (0.008)
		Non-judge	23.71 (±8.04)	23.93 (±6.39)	14	21.3 (±7.15)	23.75 (±6.13)	20	0.528 (0.009)	0.239 (0.013)	0.394 (0.007)
		Non-React	22.86 (±4.67)	20.36 (±4.62)	14	20.9 (±3.35)	23,0 (±3.95)	20	0.756 (0.002)	0.821 (0.001)	0.017 (0.075)
		Total	124.64 (±18.49)	118.57 (±14.42)	14	117.26 (±16.54)	126.89 (±20.6)	19	0.933 (≈0)	0.319 (0.007)	0.013 (0.048)
	DASS 21	Depression	6.27 (±5.61)	4.2 (±5.44)	15	6.68 (±5.32)	4.82 (±5.58)	22	0.735 (0.002)	0.065 (0.032)	0.923 (≈0)
		Anxiety	5.73 (±4.62)	3.27 (±5.11)	15	6.32 (±5.27)	5.32 (±6.21)	22	0.419 (0.015)	0.059 (0.022)	0.383 (0.005)
		Stress	6.47 (±5.21)	4.13 (±5.25)	15	6.95 (±4.82)	5.27 (±6.08)	22	0.596 (0.006)	0.048 (0.033)	0.738 (0.001)
SchoolMiSP	CAMM		30 (±7.67)	28.07 (±6.78)	73	25.02 (±7.06)	28.17 (±6.99)	41	0.022 (0.025)	0.908 (≈0)	0.008 (0.027)
	DASS 21	Depression	6.93 (±6.40)	10.48 (±7.01)	73	11.59 (±7.54)	8.24 (±6.28)	41	0.216 (0.007)	0.218 (0.006)	2.18e-04 (0.056)
		Anxiety	7.37 (±7.01)	11.08 (±7.78)	73	13.24 (±8.53)	9.46 (±7.88)	41	0.045 (0.017)	0.324 (0.004)	6.81e-04 (0.052)
		Stress	5.78 (±5.79)	8.38 (±6.38)	73	10.73 (±7.80)	7.76 (±7.17)	41	0.026 (0.024)	0.472 (0.002)	0.0016 (0.039)
University MBCT	AAQII		32.45 (±6.23)	30.9 (±8.88)	20	32.84 (±7.47)	33.16 (±6.76)	19	0.535 (0.008)	0.555 (0.001)	0.391 (0.004)
	BDI		3.3 (±2.87)	3.15 (±2.98)	20	3.32 (±3.64)	3.05 (±2.55)	19	0.963 (≈0)	0.604 (0.001)	0.886 (≈0)

*Effect sizes (η^2^g) < 0.0001 are denoted as ≈0, meaning a negligible effect.*

When assessing the impact over mental health, we found significant effects of the intervention only for Psychosis-MBCT and School-MiSP. In general, both interventions improve mental health, observed by significant and tendency (*p* < 0.1) results in Measurement (Table 2). The only impacts observed due to the mindfulness intervention, are observed in School-MiSP for depression, anxiety, and stress. Table 2 shows how depression increased in Control group, while decreased in mindfulness group. The same pattern can be observed for anxiety and stress (Table 2). Together, this results support that mindfulness interventions can be beneficial. Nonetheless, there is great variability on intervention outcomes, which motivated to evaluate how baseline scores may impact over participants’ trajectories.

### Baseline Mindfulness and Mindfulness Change

**FIGURE 1:**
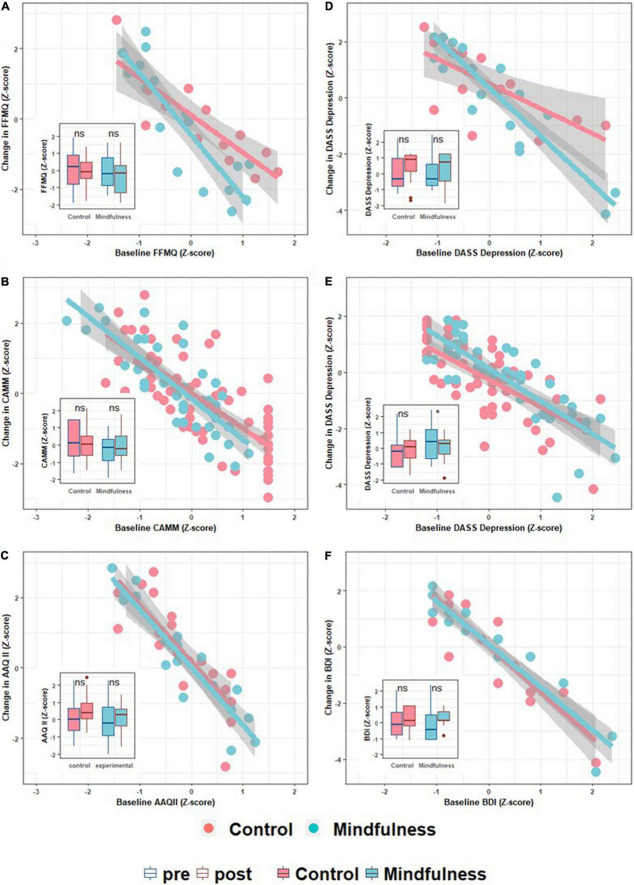
Scatter and boxplots depicting intervention effects. In the scatter plots, the *x*-axis represents the baseline value for a specific variable, while the *y*-axis represents its change (Δ) post intervention. The first column of the panel plots **(A–C)** are mindfulness-related variables, while the second **(D–F)** are psychopathology severity scores. Each row refers to a different sample. The first row is the Psychosis-MBCT sample **(A,D)**, the second row is the School-MiSP sample **(B,E)**, and the last row corresponds to the University-MBCT sample **(C,F)**. All plots are complemented by a boxplot inset depicting group pre- and post-intervention changes. This allows us to present both the participants’ trajectories (scatter plots) and the common group-based analytic approach (contrasting averages).

Globally, participants with higher baseline mindfulness scores exhibit fewer changes in Mindfulness scores (Figures 1A–C). Consistently for all RCTs and mindfulness scales used during post-intervention and follow-up, baseline mindfulness score is a significant predictor of mindfulness change (Table 3). Most regressions explained about 60–75% of the variance in all these conditions. Notably, Adjusted *R*^2^ values are high even in those models where the baseline score is the only regressor. Baseline scores alone can account for 28–76% of data variability. This suggests that baseline scores can explain a large proportion of mindfulness change variability and up to three quarters of total change variability. In global terms, results are consistent despite differences in terms of mindfulness instruments, sample characteristics, control groups, sample sizes, and moment of change assessment.

**TABLE 3:** Summary of mindfulness multiple linear regression models.

RCT	Change moment	Dependent variables	Baseline score of dependent variable	Treatment: MT	Interaction	Adjusted *R*^2^
Psychosis-MBCT	Post	Δ Observe	–1.54 (±0.19)***			**0.65**
		Δ Describe	–1.67 (±0.15)***	–0.84 (±0.27)**		**0.78**
		Δ Act Aware	–1.38 (±0.17)***			**0.65**
		Δ Non Judge	–1.72 (±0.26)***	–0.70 (±0.32)*	–0.94 (±0.34)*	**0.60**
		Δ Non react	–1.34 (±0.13)***			**0.76**
	Follow-up	Δ Observe	–1.58 (±0.19)***			**0.75**
		Δ Describe	–1.27 (±0.17)***			**0.71**
		Δ Act Aware	–1.43 (± 0.26)***			**0.52**
		Δ Non Judge	–0.91 (± 0.29)**			**0.28**
		Δ Non react	–0.85 (±0.18)***			**0.48**

School-MiSP						
	Post	Δ CAMM	–1.08 (±0.08)			**0.61**
	Follow-up	Δ CAMM	–1.25 (±0.12)***	–0.51 (±0.19)**	–0.55 (±0.19)**	**0.75**

University-MBCT						
	Post	Δ AAQ-II	–1.69 (±0.15)***			**0.75**
	Follow-up	Δ AAQ-II	–1.7 (±0.22)***			**0.70**

Regression coefficients are presented for each dependent variable. We evaluated the interactions between intervention groups and baseline scores. Blank cells represent tested variables that were removed during pruning. All dependent variables are changes obtained from the subtraction of Post/Follow-up scores from the baseline (Δ). *p < 0.05, **p < 0.01, ***p < 0.001.

For the Psychosis-MBCT sample with respect to post intervention changes, Treatment as Usual (TAU) increases about one point on the same scale compared to TAU with mindfulness. This suggests that mindfulness change is not only derived from mindfulness interventions and that, in this case, TAU was more effective in increasing this facet of Mindfulness. In the same sample, also regarding post intervention change, FFMQ Non-Judge exhibited a main effect of treatment with a significant interaction. The interaction is reflecting the different slope between TAU and TAU + Mindfulness, between baseline FFMQ and Non-judge, and between post change FFMQ and Non-judge. Participants with lower baseline scores in this FFMQ facet exhibit similar changes, while participants with high scores in the same facet exhibit higher increments in TAU compared with TAU + Mindfulness. This tendency is also observed in global FFMQ score (Figure 1A). This tendency of mindfulness treatment to reduce increases in mindfulness is also observed in the School-MiSP sample at follow-up, when Mindfulness exhibited a significant negative effect joined by a significant interaction. This interaction reflects the same effect described above, where differences in signs are due to using Mindfulness instead of control group as reference. So, independently of the sample and the moment when the treatment impacts on Mindfulness change, the Mindfulness treatment group presented less change compared to the control groups.

### Assessing Depression, Anxiety, and Stress Change

In this second step of linear modeling, we used the dependent variable baseline score as predictor of itself. For instance, we used baseline stress to predict the change in stress. We also included the treatment and the change in mindfulness scores as regressors. All regression models indicated that the baseline scores were significant (Table 4). Overall, the higher the score, the higher the reduction in such score (Figures 1D–F). Most regression models explained about 70–85% of the change in dependent variables. Including Group and change in mindfulness related variables increase the explained variance of these models 8.7% on average (SD: 5.9), with a maximum of 19% and a minimum of 1%, meaning that baseline scores explain far more variability than intervention itself. The effect of baseline scores was consistent regardless of sample characteristics, control group differences, sample sizes, and moment of change assessment.

**TABLE 4:** Summary of mental health multiple linear regression models.

RCT	Change moment	DV	Baseline score of DV	Δ Observe	Δ Describe	Δ Act Aware	Δ Non-judge	Δ Non-react	Treatment: MT	Interaction	Adjusted *R*^2^ (M.H.)	Adjusted *R*^2^ (M.H. and Mind.)
Psychosis-MBCT	Post	Δ Stress	–1.65 (±0.18)***	–0.24 (±0.10)*							**0.66**	**0.72**
		Δ Depression	–1.49 (± 0.16)***	–0.17 (±0.08)*							**0.66**	**0.72**
		Δ Anxiety	–1.54 (±0.15)***			–0.27 (±0.10)*					**0.75**	**0.80**
	Follow-up	Δ Stress	–1.33 (±0.20)***								**0.67**	**NA**
		Δ Depression	–2.15 (±0.20)***			0.20 (±0.08)*			–0.48 (±0.28)	–1.13 (±0.28)***	**0.72**	**0.85**
		Δ Anxiety	–1.35 (0.13)***								**0.81**	**NA**

			**Baseline score of DV**	**Δ CAMM**					**Treatment: MT**	**Interaction**		

School-MiSP	Post	Δ Stress	–0.60 (±0.11)***	–0.47 (±0.06)***	NA	NA	NA	NA	0.24 (0.15)	–0.31 (±0.15)*	**0.56**	**0.72**
		Δ Depression	–0.77 (±0.09)***	–0.41 (±0.06)***	NA	NA	NA	NA	0.42 (±0.16)*		**0.53**	**0.65**
		Δ Anxiety	–0.52 (±0.11)***	–0.47 (±0.06)***	NA	NA	NA	NA	0.29 (±0.16)	–0.31 (±0.15)*	**0.48**	**0.67**
	Follow-up	Δ Stress	–0.95 (0.12)***	–0.32 (±0.06)***	NA	NA	NA	NA	–0.05 (±0.15)	–0.31 (±0.14)*	**0.81**	**0.86**
		Δ Depression	–1.16 (±0.10)***	–0.24 (±0.06)***	NA	NA	NA	NA			**0.77**	**0.81**
		Δ Anxiety	–1.22 (0.10)***	–0.17 (±0.06)**	NA	NA	NA	NA			**0.81**	**0.82**

			**Baseline score of DV**	**Δ AAQ-II**					**Treatment: MT**	**Interaction**		

University-MBCT	Post	Δ BDI	–1.5 (±0.10)***		NA	NA	NA	NA			**0.86**	**NA**
	Follow-up	Δ BDI	–1.57 (±0.12)***		NA	NA	NA	NA			**0.87**	**NA**

Regression coefficients are presented for each dependent variable. We evaluated the interactions between intervention groups and baseline scores. Blank cells represent tested variables that were removed during pruning. NA stands for does not apply, meaning that those variables were not tested. All dependent variables are changes obtained from the subtraction of Post/Follow-up scores from the baseline (Δ). In this case, mindfulness predictors are also changes (Δ) produced in the same period of time as the one used in dependent variables. Two adjusted R2, where M.H. stands for baseline of mental health variables as predictors, and M.H. and Mind. stands for R2 including changes in mindfulness and Group as predictors. *p < 0.05, **p < 0.01, ***p < 0.001.

Regarding the contributions of mindfulness change, in all School-MiSP’s models, at post-intervention and follow-up, it had a positive effect reducing the scores. The only exception was the University-MBCT sample, which did not exhibit any significant differences that can be attributed to the change in the AAQ-II. Importantly, many of the regression models which revealed a main effect of mindfulness change also had interactions. These main effects can be interpreted as population average differences, while the interaction indicates a difference in slopes. Thus, a negative interaction means that MT treatments resulted in a stronger association between baseline scores and change in those scores compared to the Control group, meaning that participants exhibited more MT change even when controlling for baseline.

For the Psychosis-MBCT at Post-intervention, FFMQ observe had a beneficial effect on stress and depression change after controlling for the baseline scores of both. For anxiety, FFMQ act aware exhibited a beneficial effect. At Follow-up, only depression had an effect besides baseline scores, along with treatment (with an interaction effect). For the School-MiSP sample, we found that a change in CAMM was a significant predictor of post intervention changes in stress, depression, and anxiety. Only depression did not present an interaction, supporting the view that the effect of baseline depression scores on post-intervention depression scores was independent of the intervention conducted. Importantly, the CAMM change during the interventions only predicted stress change at follow-up after controlling for stress baseline scores.

## Discussion

The results presented in this article highlight the critical role of the participants’ pre-MBI status. Our results indicate that at least two thirds of the change produced by these interventions in terms of mindfulness scores and depression, stress, and anxiety can be predicted by the baseline scores of the same variables. We also found in some subscales that trajectories are not only strongly influenced by the initial status of the participants, but also by the intervention performed, as seen in the significant interactions found. This results support the relevance of considering baseline scores as key elements to understand the individual trajectories of participants, in contrast with only considering population proxies such as average change.

Even more interesting, is the fact that classic approach did not present consistent results with the linear regression models. This means, that once the major source of variance is controlled (initial status of the participant), then treatment efficacy should be addressed. For instance, FFMQ’s observe subscale presented a significant interaction suggesting an impact of both, Group and Measurement (Table 2). However, Treatment was not a significant regressor when controlling by baseline scores of FFMQ’s observe subscale (Table 3). Conversely, FFMQ’s non-judge subscale presented no effect by means of the mindfulness intervention using the classic approach, but it did present a significant contribution when considering the baseline scores of participants. As such, assessing the baseline scores is not only a matter of considering participants’ trajectories, but also to control adequately confounding variables which may explain RCT outcomes. This means that considering baseline scores represent a methodological concern as well.

Considering that we only assessed the baseline scores of the variables that we wanted to predict (i.e., Mindfulness, depression, anxiety, and stress), it is critical to consider other potential aspects which may have driven participants’ trajectories. For instance, mindfulness has been positively associated with positive affectivity and conscientiousness, and inversely related to neuroticism and negative affectivity (Borynski, 2007; Fetterman et al., 2010). Lee and Bowen (2015) found that four of the five personality factors of the Toronto Mindfulness Scale (TMS) (Lau et al., 2006) (i.e., Conscientiousness, Extraversion, Agreeableness, and Neuroticism) were significantly associated with Decentering of mindfulness at baseline. Even attachment style has been proposed as a relevant element of participants’ trajectories (Cordon et al., 2009). Despite this evidence, interventions are designed and applied without considering how inter-individual differences may play a critical role in patients’ trajectories during an intervention.

### Participants’ Trajectories

One of the possible reasons behind the neglect of trajectories in the literature is the traditional way in which RCTs are analyzed. In Figure 1, we included as insert the traditional plot which presents the before and after scores divided by intervention. As Figure 1 show, it is not possible to infer the association presented in the scatter plots. The main reason is that we assume that an eventual global drop in a boxplot is due to fairly similar changes in all the participants. This bias has been methodologically challenged and considered to be a limitation of the classic mixed ANOVA approach (Barr et al., 2013). Our results, in contrast, suggest that some participants exhibit huge changes while others show minor ones, with some even undergoing negative changes (i.e., undesired effects). Therefore, the traditional results report neglects individual trajectories, masking the diversity of trajectories found across participants.

Overall, our results indicate that the initial status of participants is critical to the outcome, regardless of the population sampled and the mindfulness instrument used. In general terms, the participants who had the lowest baseline mindfulness scores improved the most. Similarly, those with the highest stress, depression, and anxiety scores showed the highest drops in symptomatology. These results reflect a room for change phenomenon, as people with the most severe symptoms or the lowest mindfulness scores, depending on the score analyzed, have more room for improvement. Paradoxically, these improvements may lead them to outperform people who started in better conditions. Even more critically, those who start with the lowest symptomatology (or highest mindfulness scores), may even worsen their scores. Importantly, this is a description of what we observed on the results, the actual causes of how and why occurs this phenomenon still required further research to be understand and use in favor of better interventions.

Mindfulness interventions have been widely reported to be beneficial (Shonin et al., 2013; Khoury et al., 2015; Tang et al., 2015; Carsley et al., 2018; Steinebach and Langer, 2019) therefore, our analytic approach should capture these population-level effects which neglect individual trajectories. Under our analytic approach, which considers baseline scores, the population impact of mindfulness, regardless of individual trajectories, is mainly depicted through interactions. These interactions allow us to model a differential slope between mindfulness and control groups (hence the population rationale) with respect to the relation between baseline scores and changes in post-intervention scores. Our results support prior evidence for the benefits of mindfulness, as we found for some subscales linear regression Group effects’ and interactions indicating that the trajectory of participants was more beneficial in the mindfulness group compared to controls. This means that, as group average, mindfulness interventions are likely better than the controls. Nonetheless, mindfulness interventions presented a minor impact over the variability of mental health change post intervention (8.7% ± 5.6 of explained variance difference), indicating low benefits.

### External Validity: Samples, Instruments, and Interaction

We are not the first group to evaluate the potential contribution of baseline scores to intervention outcomes in RCTs (Shapiro et al., 2011; Tortella-Feliu et al., 2020). The novelty of this study is that it evaluates the role of baseline mindfulness considering interactions while also viewing mindfulness change during interventions as a trigger of their benefits. We also used different populations and different mindfulness instruments. Considering the interaction with treatment, as above mentioned, was relevant to reveal the differences between groups outcomes. This allowed us to detect the widely reported beneficial effect of mindfulness while also including other predictors to tackle individual differences in participants’ trajectories. Changes in mindfulness were also a relevant predictor of changes in symptomatology, indicating that it is the trajectory rather than the raw score that we should take into account when trying to predict participants’ improvement.

The most relevant difference across the samples used was found in the University-MBCT sample, which used the AAQ-II. In this sample, we observed no relevant contributions of AAQ-II change to BDI change at post-intervention and follow-up. Thus, for this sample, changes during the intervention cannot be attributed to changes in the AAQ-II. In this regard, it is relevant to consider that, despite the widespread use of the AAQ-II as a measure of experiential avoidance (EA) which correlates with mindfulness (Fledderus et al., 2012), EA has been conceptualized as the opposite of acceptance. Nevertheless, it has been criticized on the grounds that it does not adequately discriminate constructs like neuroticism or general distress (Valencia, 2019).

Despite this difference with the AAQ-II, our results, and those found previously (Shapiro et al., 2011; Harnett et al., 2016; Tortella-Feliu et al., 2020), can most likely be generalized regardless of the sample characteristics and the instrument used to measure mindfulness. Our results support the view that an instrument more closely related to the global construct of mindfulness than the AAQ-II is required to obtain these results. Critically, the impact of baseline scores on trajectory was found in a range of samples from large (such as our School-MiSP sample) to small [such as that of Tortella-Feliu et al. (2020) or our Psychosis-MBCT sample]. In fact, when observing Figure 1, the small amount of noise on this association is quite evident, which is also reflected in high adjusted R2 values (Tables 3, 4).

### Considering Trajectories During Intervention Planning

Our results support that neglecting initial status of participants is a relevant source of variability which impacts the outcome of intervention. The interventions presented here were beneficial for the population, however, results support that using averages kept us blind to individual phenomena relevant for the outcomes. Even more, it presents how artifactual results can be obtained if ignoring initial participants status. Given these results, it is mandatory to consider individual trajectories at least using initial status of the participants.

Concretely, our results have direct implications for interventions in clinical and non-clinical contexts. In this regard, given that the Mindfulness trait has been reported to be relatively stable (Rau and Williams, 2016), it is relevant to assess dispositional mindfulness as a part of the standard application of MBIs as well as other psychosocial interventions that have shown to increase mindfulness skills (Xia et al., 2019). Thus, group set up criteria should not only consider participants’ diagnosis but mainly their dispositional mindfulness and the severity of their symptomatology.

The above considerations can help to generate a tailored intervention while also yielding relevant information for the instructor to guide the intervention group. This can allow the instructor to keep a steady pace by supporting the trajectories of participants with a homogeneous level of mindfulness skills and symptomatology severity. Otherwise, the progress of participants with higher skills and low symptomatology could be diluted, eventually causing them to drop out of the intervention.

In this regard, given our results, it is questionable whether the frequency and duration of mindfulness interventions can be standard for all participants. Thus, short interventions may be appropriate for people with high dispositional mindfulness and mild symptomatology (Howarth et al., 2019), while vulnerable or specific populations may require more support (Langer et al., 2020).

### Limitations

Despite the many virtues of our samples from three different RCTs, the procedure presented in this article also has some relevant caveats. The most obvious one is that our results are derived from experimental designs which were not produced to evaluate the impact of baseline status on intervention outcomes. In consequence, there is no proper follow-up during and after the intervention, besides the usual approach. For instance, we do not know if people who had high mindfulness and whose score dropped post-intervention actually experienced a worsening of their situation or if they realized that they were not as mindful as they initially reported. We lack qualitative data supporting insights into these trajectories, which limits the potential measures to be taken in future interventions. Therefore, we advise caution when implementing the suggestions presented above. Our results do support the view that intervention participants have diverse trajectories; however, more research and close follow-up is required to produce exhaustive and safe recommendations capable of informing a personalized approach. Apart from these aspects, the heterogeneity of our samples is a virtue in terms of external validity. Nonetheless, our results are not exactly the same for all the samples, and the reasons for minor discrepancies are obscured by differences in sample characteristics and size. This introduces interpretative and statistical noise which limits the scope of our conclusions. Despite these limitations, this study confirms the need to continue doing research in a way that considers the diversity of participants’ trajectories, expanding the room we have for intervention improvements informed by a more personalized approach to treatment.

## Data Availability Statement

The original contributions presented in this study are included in the article/supplementary material, further inquiries can be directed to the corresponding author/s.

## Ethics Statement

The studies involving human participants were reviewed and approved by the University-MBCT (Doctoral thesis AIL; research group HUM 760, Almeria University), School-MiSP (project no 82130055: Faculty of Psychology of the Pontificia Universidad Católica de Chile), Psychosis-MBCT (project no 11150846; National Health Service in Valdivia). The patients/participants provided their written informed consent to participate in this study.

## Author Contributions

RV and ÁL: conceptualization. RV: methodology and formal analysis. EL-P: data curation. RV, CB, and ÁL: writing—original draft preparation. RV, CB, EL-P, CS, and ÁL: writing—review and editing. CS: funding acquisition. All authors have read and agreed to the published version of the manuscript.

## Conflict of Interest

The authors declare that the research was conducted in the absence of any commercial or financial relationships that could be construed as a potential conflict of interest.

## Publisher’s Note

All claims expressed in this article are solely those of the authors and do not necessarily represent those of their affiliated organizations, or those of the publisher, the editors and the reviewers. Any product that may be evaluated in this article, or claim that may be made by its manufacturer, is not guaranteed or endorsed by the publisher.
